# Integrating the tumor-suppressive activity of Maspin with p53 in retuning the epithelial homeostasis: A working hypothesis and applicable prospects

**DOI:** 10.3389/fonc.2022.1037794

**Published:** 2022-11-29

**Authors:** Sijie Tang, Zhongli Ling, Jiajia Jiang, Xiang Gu, Yuzhong Leng, Chaohui Wei, Huiying Cheng, Xiaohua Li

**Affiliations:** ^1^ Department of Urology, Affiliated Aoyang Hospital of Jiangsu University, Zhangjiagang, Suzhou, China; ^2^ Aoyang Cancer Institute, Affiliated Aoyang Hospital of Jiangsu University, Zhangjiagang, Suzhou, China

**Keywords:** tumor suppressive Maspin, p53, epigenetic regulation, epithelial homeostasis, immune surveillance

## Abstract

Epithelial malignant transformation and tumorous development were believed to be closely associated with the loss of its microenvironment integrity and homeostasis. The tumor-suppressive molecules Maspin and p53 were demonstrated to play a crucial role in body epithelial and immune homeostasis. Downregulation of Maspin and mutation of p53 were frequently associated with malignant transformation and poor prognosis in various human cancers. In this review, we focused on summarizing the progress of the molecular network of Maspin in studying epithelial tumorous development and its response to clinic treatment and try to clarify the underlying antitumor mechanism. Notably, Maspin expression was reported to be transcriptionally activated by p53, and the transcriptional activity of p53 was demonstrated to be enhanced by its acetylation through inhibition of HDAC1. As an endogenous inhibitor of HDAC1, Maspin possibly potentiates the transcriptional activity of p53 by acetylating the p53 protein. Hereby, it could form a “self-propelling” antitumor mechanism. Thus, we summarized that, upon stimulation of cellular stress and by integrating with p53, the aroused Maspin played the epigenetic surveillant role to prevent the epithelial digressional process and retune the epithelial homeostasis, which is involved in activating host immune surveillance, regulating the inflammatory factors, and fine-tuning its associated cell signaling pathways. Consequentially, in a normal physiological condition, activation of the above “self-propelling” antitumor mechanism of Maspin and p53 could reduce cellular stress (e.g., chronic infection/inflammation, oxidative stress, transformation) effectively and achieve cancer prevention. Meanwhile, designing a strategy of mimicking Maspin’s epigenetic regulation activity with integrating p53 tumor-suppressive activity could enhance the chemotherapy efficacy theoretically in a pathological condition of cancer.

## Characteristics of Maspin

Maspin (mammary serine protease inhibitor) was originally identified by utilizing subtractive hybridization analysis in breast epithelial materials and was reported to specifically express in epithelial tissues (1, 2). Later on, it was found that Maspin played a crucial biological role in early embryonic development since knockout of Maspin in mice is embryonically lethal (2). A structural homology study revealed that Maspin belonged to the serine protease inhibitor (serpin) superfamily with an existing typical reactive center loop (RCL) motif in its protein conformational structure, and this RCL is an active region and serves as a decoy for a targeted molecule to execute its biological functions (3). The 3D spatial structure of the Maspin protein contains three β-sheets, nine α-helices and the hydrophobic RCL lies closer to the serpin core of the molecule. It was believed that the presence of the RCL is the main molecular structural feature for the antitumor properties of native Maspin (4, 5). The existence of a G-helix structure is an internal salt bridge of the RCL. A study showed that aberrant changes in the G-helix structure impaired performance of Maspin in inhibiting cell migration and affecting cell adhesion (6).

As a tumor-suppressive molecule, Maspin has been shown to prevent chronic inflammation; inhibit tumor cell invasion, migration, and angiogenesis; arrest pathologic cell-cycle progress; induce malignant cell undergoing re-differentiation; and sensitize tumor cells to drug treatment (7–11). Numerous studies have shown the relevance of an abnormal expression of Maspin in epithelial cells with malignant transformation as well as cancer development and progress, e.g., the loss of Maspin expression conferred the gain of ability in boosting tumor progression in many types of cancer.

In addition, the antitumor effect of Maspin is also closely related to its subcellular localization. Maspin is mainly present in the cytoplasm but also in the cell membrane, secretory vesicles, mitochondria, and nucleus in malignant tissue cells (12). Accumulated evidences showed that the nuclear localization of Maspin favored tumor prognosis. Meanwhile, a study revealed that the nuclear localization of Maspin could be regulated by calcium-dependent cell–cell contact, EGFR, JAK-STAT3 and PI3K-Akt pathways (13). Thus, Maspin may appear variant antitumor activities in different types or progress stages of tumor.

Several recent studies also highlighted that Maspin was associated with variant epigenetic regulatory networks *via* the interplaying between DNA methylation and histone posttranslational modifications (7, 14, 15). It was notable that aberrant histone deacetylase (HDAC) expression and activity were found in diverse human cancers, which was correlated with abnormal epigenetic modification of certain important genes along with the progress/prognosis of disease (16, 17). For instance, a high expression of class I HDACs including HDAC1 was reported in prostate cancer tissue, and HDAC1 is the most abundant class I HDAC in mammalian cells. Maspin was evidenced to be an endogenous HDAC1 inhibitor in prostate epithelial cells to epigenetically regulate certain gene expressions, which contribute to or favor its tumor-suppressing activities (10, 18).

It was also found that the expression of Maspin was associated with better differentiation phenotypes of human cancer tissues, and an increase in Maspin expression in cancer cells could restore the epithelial re-differentiation characteristics, which could be endorsed through this Maspin-mediated epigenetic regulation machinery (19, 20). Clinical studies also found that Maspin expression was significantly correlated with the overall survival (OS) and progression-free survival (PFS) rates in muscle invasive bladder cancer (MIBC) patients who received cisplatin-based neoadjuvant chemotherapy (NACT) (21). Indeed, patients with reduced or absent Maspin expression showed a lower rate of both their OS and PFS rates than those with Maspin expression. Meanwhile, increasing Maspin expression sensitized tumor cells to the treatment with cisplatin along with the reduction of PI3K/AKT/mTOR signaling activity in bladder cancer (21). Maspin in oral squamous cell carcinoma (SCC) was also reported to be associated with a better prognosis (22). In addition, Maspin was found to possibly function as a relevant inhibitor to prevent the local invasiveness and further systemic progression of prostate cancer (23).

As an epithelial biomarker, Maspin was considered to play a critical role in fine-tuning epithelial homeostasis (11). Since recent evidence uncovered the existence of a complicated interactive molecular network of Maspin (e.g., p53, GSTpi, AR) (9, 24, 25), further sorting and clarifying this network is desirable for continually gaining insights on its cellular and biological functions toward epithelial differentiation and homeostasis. In our opinion, Maspin seems more like a “guardian of the epithelium” through its epigenetic surveillant role in retuning epithelial homeostasis.

## Maspin-mediated antitumor activity

It was studied that the lack of Maspin in lung adenocarcinoma (LUAD) was associated with the poor phenotypes of tumor including lymph node metastasis, late TNM stage, bad prognosis, and recurrence (26). Patients with higher Maspin levels were observed to have significantly better prognosis. In addition, Katakura et al. reported that the 5-year overall survival rates were 67.7% for patients with a higher Maspin expression and 41.4% for those with a lower Maspin level (27). Furthermore, nuclear localization of Maspin appeared to suppress the LUAD cell invasion, whereas cytoplasmic Maspin promoted it (28).

**Figure 1:**
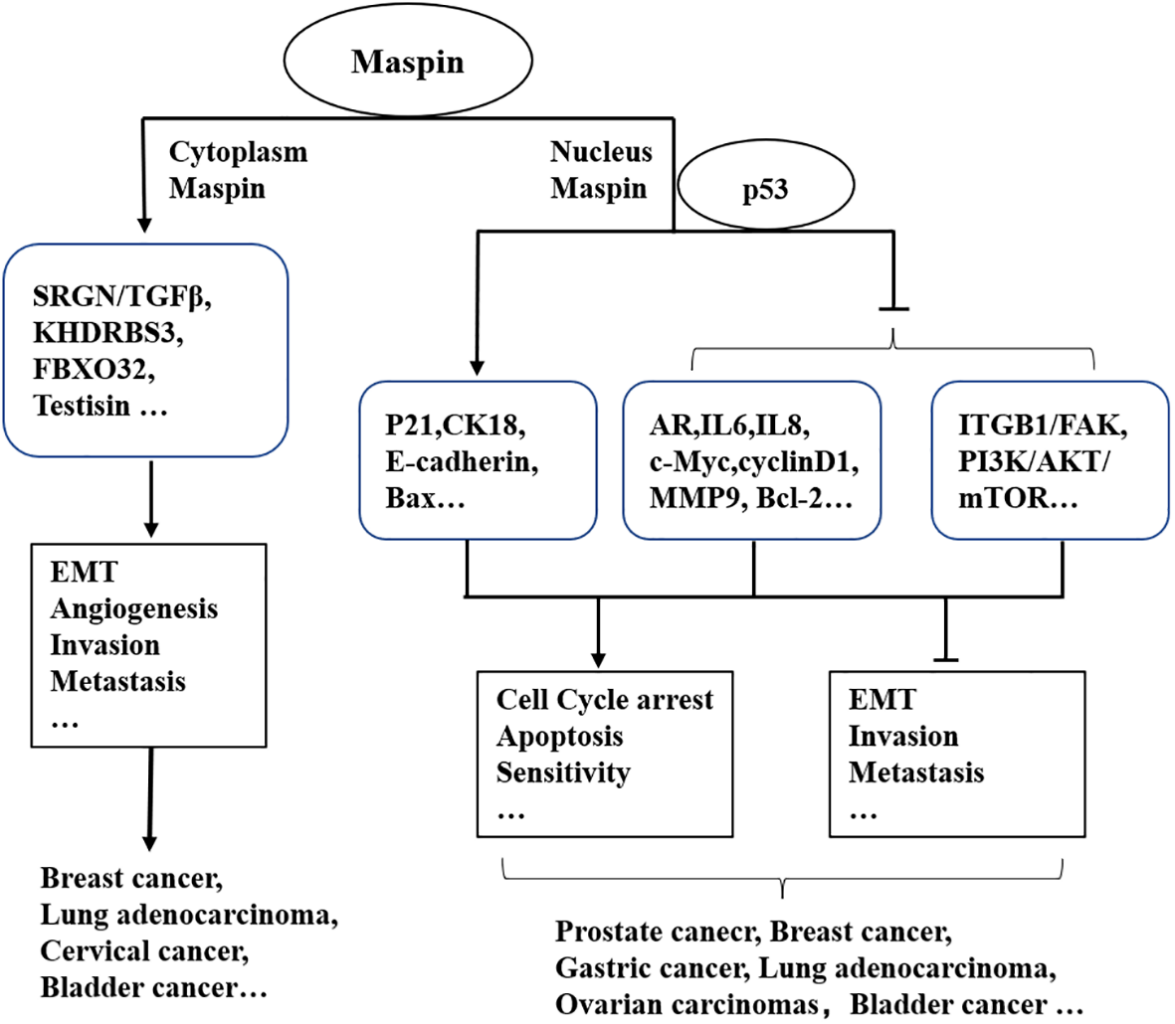
Maspin-associated signaling alteration in cellular events.

Similar phenomena were also reported in other cancers (**Figure 1**). Nuclear expression of Maspin in 132 invasive epithelial ovarian carcinoma patients confirmed by immunocytochemical assay was correlated with a good prognostic factor and a better long-term survival (29). However, cytoplasmic localization of Maspin promoted breast cancer cell invasion and metastasis *via* activating the SRGN/TGFβ signaling axis (30). Uchinaka et al. also found that the expression of Maspin in the cytoplasm alone implied an unfavorable prognosis in patients with pancreatic ductal adenocarcinoma (PDAC) (31). Given the most common cancers, such as prostate cancer and breast cancer, the antitumor effect of Maspin is beyond doubt. Maspin was also reported to inhibit gastric cancer (GC) cell invasion and migration by blocking the cellular ITGB1/FAK signaling pathway and in turn reducing epithelial–mesenchymal transition (EMT) and angiogenesis (32, 33). A study found that GC tissues with a low expression of Maspin were usually associated with a low expression of E-cadherin and a high expression of vimentin (34). However, it was also observed that a high expression of Maspin might promote the progression of GC (35). For example, a high Maspin expression was found in a subtype of adenocarcinoma (AC) rather than in non-small cell lung cancer (SCLC) and is related with a higher TNM stage in AC (36).

The occurrence and development of tumors could be a consequence of the imbalance of cells in proliferation and differentiation, which could also be genetically connected to the activation of some oncogenes or the inactivation of tumor-suppressor genes. As a tumor-suppressive molecule of Maspin, its suppression, deletion and mutation were closely related to malignant transformation and tumorigenesis. Studies demonstrated that the molecule of Snail could directly inhibit Maspin promoter activity and resulted in a decrease in Maspin expression and an increase in cell migration and invasion. Chromatin remodeling complex CBP/p300 could decrease the binding of Ets-1 and c-Jun to the Maspin promoter, subsequently lead to the deficiency of Maspin, which were accompanied by the appearance of malignant phenotypes (14, 37–39).

It was reported that both high deacetylation in histone lysine residue and hypermethylation of CpG islands of the Maspin promoter region silenced Maspin expression (7, 40). Our previous study also showed a role of Maspin in regulation of HDAC-targeted genes including Bax, cytokeratin 18 (CK18), and p21(WAF1/CIP1) (41). The DNA methyltransferase inhibitor 5-aza-2′-deoxycytidine (5-aza-CdR) and/or the HDAC inhibitor Trichostatin A (TSA) strongly upregulated the expression of Maspin in breast cancer and prostate cancer cells (42, 43). An *in vitro* study revealed that procyanidin B2, a non-toxic compound in nature, could be a potent inhibitor of DNA methyltransferases, coming up with reactivation of E-cadherin, Maspin, and BRCA1 gene at the transcription level (44). Specifically designed artificial transcription factors (ATFs) can reactivate Maspin expression in cancer cells and in turn benefit in cancer prognosis. This strategy could be a potential treatment application for a wide range of Maspin-defected diseases (45).

In prostate cancer, abnormal activation of androgen receptor (AR) signaling is the essential characteristic for cancerous cells to maintain survival and to proliferate continually. It was reported that AR was recognized and bound to AR elements of a Maspin promoter in tumor cells which negatively regulated Maspin expression (46, 47), but this phenomenon was not confirmed by reexpressing AR in a PC3 cancer cell line. In turn, our recent study found that Maspin negatively regulated the transcription and expression of AR and synergistically enhanced the antitumor effect of enzalutamide (9). Therefore, clarifying the underlying mechanism of the interactive network of Maspin is particularly important for the prevention and targeting treatment of prostate cancer. Further investigation is also needed to uncover the clinical significance of Maspin in negative regulation of AR pathways.

## Maspin in guiding epithelial development

An appropriate dynamic movement between differentiation and proliferation is important to guide normal epithelial development, which should be precisely regulated by certain factors and/or mechanisms to guard and maintain this physiological epithelial homeostasis. Breaking this homeostasis led to either senescence or cancerous growth. EMT is a necessary event of epithelial malignant transformation toward cancerous growth. It was well known that EMT could be characterized by loss of the epithelial hallmarks with increase of mesenchymal phenotypes, malignant transformation and tumorigenesis. Maspin expression was found to be positively correlated with E-cadherin expression but negatively correlated with the expression of vimentin in GC tissues. A study showed an important correlation between Maspin and EMT (34). Several EMT-related transcription factors are regulated by HDACs to maintain protein stability (48). By inhibiting HDAC activity, the tumor-suppressor Maspin and its mediated epigenetic changes facilitated to reprogram tumor cells toward a better-differentiated phenotype by targeting the p-Stat3/c-Myc signaling pathway and reducing inflammatory IL-6 and IL-8 (49). Also, Maspin regulated a set of HDAC1-targeted genes, which could be involved in the process of epithelial differentiation and cellular stress-induced mesenchymal-to-epithelial transition (MET) (19, 25). Thus, Maspin appeared to be partially responsible for preventing or even reversing the EMT processes.

When cells undergo EMT, cell signaling pathways and cell cytoskeleton dynamically change (50). Indeed, quantification of cell morphology from an image analysis of the actin cytoskeleton revealed that Maspin could reduce cell migration and alter cytoskeletal morphology and the G-helix was proved to be the pivotal bioactive part of the molecule Maspin (51). This was consistent with the reported results that the G-helix peptide can mimic the effect of full-length Maspin and reduce the cell migration through targeting the actin cytoskeleton (6).

Evidentially, dysfunction of Maspin through deletion of exon 4 of the gene impaired epithelial differentiation and resulted in organ- and cell-type-specific atrophy, adenoma, hyperplasia and carcinoma in Maspin-*KO* mice (52).

Thus, it could be speculated that, as an endogenous HDAC1 inhibitor, Maspin may function as an epigenetic “guardian” to alter integrally the cytoskeleton *via* regulating the chromatin accessibility of transcription factors, and inhibit the EMT progression.

In addition, the notable biologic and antitumor activity of Maspin is to sensitize the malignant cell to drug-induced apoptosis, which has been approved broadly in the field (53–55). Moreover, this Maspin-sensitized cell death induction is currently emerging as a hot topic in the development of its clinical application. Furthermore, it was reported that reexpression of Maspin in DU145 cells decreased cellular autophagy but increased cell senescence (56). Thus, from reviewing the aspect of dynamic movement of epithelial development, Maspin appeared to be obviously involved in the maintenance of epithelial homeostasis and to prevent digressional processes.

## Maspin in regulating host immune homeostasis

In view of a systemic immune disease of cancer, the mechanisms of linking abnormal immunity with tumorigenesis and tumor development are highly relevant. The tumor ecosystem is composed of tumor, stromal, and infiltrated immune cells. For instance, tumor-associated macrophages showed that a tissue-specific and the subtype of macrophages in tumor tissues and microenvironment are associated with variant clinical outcomes in response to treatment. Thus, cancer immunotherapy has become the mainstay contemporarily in clinical oncology (57, 58). The development of cancer is often accompanied by a gradual decrease of both host immune surveillance and immune response. In addition, immunosuppressive environments may be formatted by accumulating immunosuppressive elements and cytokines, e.g., interleukin 10 (IL-10) and transforming growth factor beta (TGF-β) (59). The dysfunction of effective CD8+ T cells was caused by the immunosuppressive TME, including tumor-associated macrophages M2, IL-10, TGF-β, and inhibitory checkpoint signaling pathways (60), leading to the failure of CD8+ T cells recognizing and eradicating transformed malignant cells in an antigen-specific manner (61).

Maspin may play an important role in tumor-evoked host-immune response (62). It was reported that Maspin may function as an immune system modulator in the tumor microenvironment (20, 63, 64). Evidence showed that HLA-Cw6(+) psoriasis displayed greater Maspin expression along with higher levels of CD8+ T-cell proliferation. Mice with Maspin-expressing prostate cancer xenograft had an increased expression of Maspin-specific immunoglobulin G (IgG), and Maspin-expressing tumors could induce neutrophil infiltration with increased systemic and intratumoral neutrophil maturation, implying that Maspin elicits neutrophils and B cells for the host immunity to benefit tumor clearance. Thus, Maspin is believed to be able to stimulate both cellular and humoral host antitumor immune responses (20).

Neutrophils have been shown to be the first responders to be taken up at the tumor site, and their activation is often associated with extracellular fibrosis. Girish et al. found that cellular concentrations of aspirin-restored NO led to increased Maspin synthesis in neutrophils (65). It is worthy to mention that neutrophils without an estrogen receptor (ER) will not produce any Maspin when treated with estrogen (66). Meanwhile, when treated with estrogen, ER-positive breast cancer could reactivate the expression of Maspin and have a better prognosis compared with ER-invisible breast cancer patients.

It was reported that infiltration of regulatory T cells (Tregs) in tumor tissue may promote tumor progression through suppressing antitumor-immune responses (67). ErbB2 can play a substantial role in metastatic breast cancer, and its function is associated with CD4+CD25+ Tregs and is accompanied by production of the receptor activator for nuclear factor-κB ligand (RANKL). These tumor-infiltrating Tregs can be reduced by ectopic reexpression of Maspin, which is suppressed by activation of the RANKL-RANK signaling pathway (68).

It was also reported that treatment of macrophages with recombinant Maspin significantly increased the production of IL-1, TNF, IFN, IL-6, IL-12, IL-10 and the M1 marker molecule iNOS, but inhibited the expression of TGF and M2 marker molecule Arg-1 and slightly reduced phagocytic activity. Thus, it is possible to propose that Maspin might affect the tumor microenvironment by modulating macrophages in their secretion of inflammatory cytokines and in turn to facilitate macrophage M1 polarization, which suggested a potential value of Maspin in antitumor immunity therapy (63).

**Figure 2:**
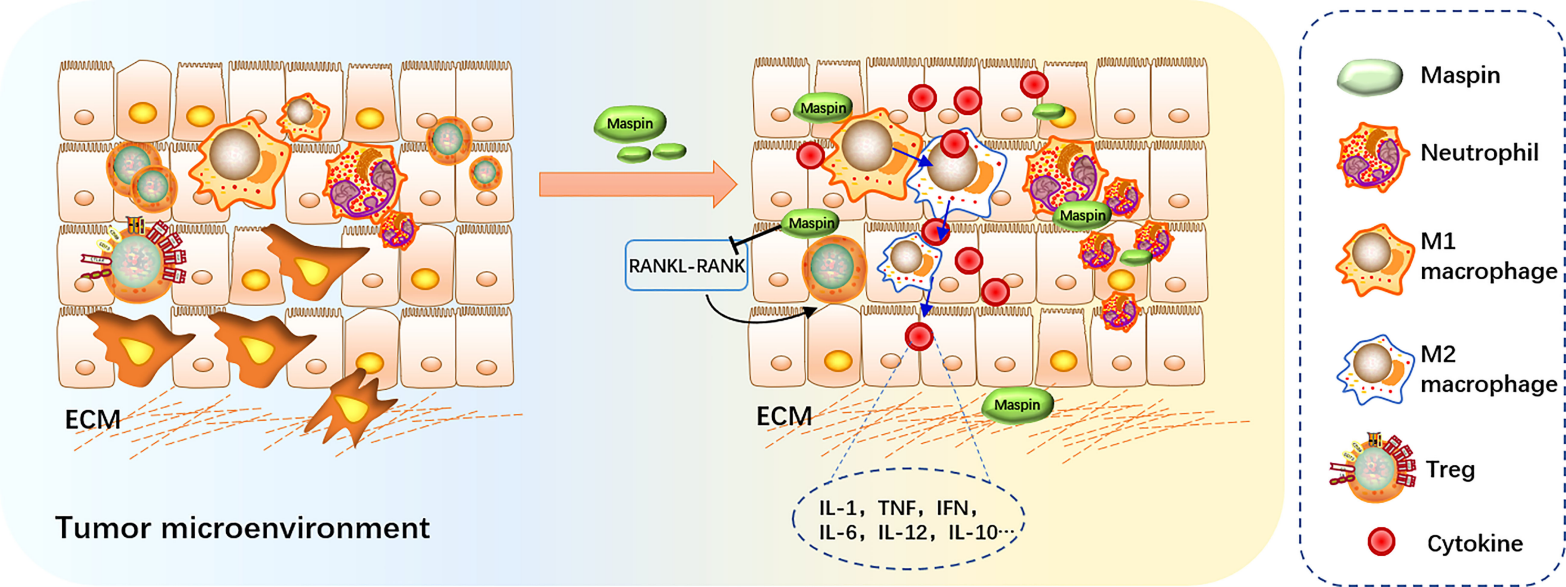
Maspin in regulating host immune surveillance. Maspin-expressing tumors induced neutrophil infiltration and macrophage M1 polarization, inhibiting tumor-infiltrating Tregs by activating the RANKL-RANK signaling pathway. Maspin contributes to remodeling the tumor microenvironment by regulating the macrophage secretion of inflammatory cytokines.

Based on the above results, Maspin may play a crucial surveillant role in directing the host immune system to eliminate the malignant transformed cancerous cells and function as another “guardian” in retuning host immune homeostasis (**Figure 2**).

## Integration of Maspin and p53 by HDAC1 in fine-tuning epithelial homeostasis

Maspin has a uniform expression in basal cells of normal and benign prostate tissues, whereas its expression remains relatively lower in malignant epithelial cells. Later on, Maspin was documented as an endogenous inhibitor of HDAC1 and resulted in HDAC1-targeted gene expression and activation through its epigenetic modification capability (41). As an endogenous HDAC1 inhibitor, Maspin’s antitumor effect is, at least partly, associated with its ability to inhibit HDAC activity. The lysine residue status of chromatin histone can be enzymatically acetylated or deacetylated, which plays an important role in switching between open and close histone-backbone structures of the chromosome. High levels of HDAC1 expression and activity were reported in cancer (17). Thus, as an HDAC inhibitor, Maspin enables retuning and reestablishing cellular acetylation homeostasis theoretically which affect certain gene expressions, e.g., reactivating the expression of tumor suppressors (18).

Meanwhile, the expression of Maspin was also under the regulation of epigenetic modification, e.g., histone acetylation/deacetylation and DNA methylation. Administration of TSA and MS-275 could significantly augment Maspin expression in prostate cancer. Thus, it could be speculated that there exists a “self-propelling” antitumor mechanism when utilizing HDAC inhibitor treatment or deigning a Maspin-based strategy in cancer therapy. It was also reported that both Maspin and HDAC inhibitor targeted a certain panel of gene expression, including upregulating p21 but downregulating cyclin D1, MMP9 and vimentin *in vitro* and *in vivo (* 10). For instance, thymoquinone (TQ), which is a bioactive phytoconstituent, could act as an HDAC inhibitor to upregulate p21 and Maspin expression, trigger pro-apoptotic gene Bax, and inhibit anti-apoptotic gene Bcl-2 in breast cancer (69).

Loss of p53, as “guardian of the genome,” by TP53 gene mutations was found in around half of all cancers and is the gateway to genetic chaos. Maspin was evidenced to be the downstream factor of p53 in its inhibition or prevention of cancer development and progression. A gel shift assay revealed that p53 activated Maspin expression by binding directly to the p53 consensus-binding site presented in the Maspin promoter (70, 71). Further study showed that mutations in exon 7 of the p53 gene are responsible for downregulating Maspin, but wild-type p53 showed a partial restoration of nuclear Maspin expression (72). Silencing class I HDACs could facilitate the binding of p53 to the Maspin promoter (7, 70). Another study also reported that repression of HDAC1 and HDAC8 activated Maspin transcription along with significant enrichment of p53 at the Maspin promoter and increased histone H3/H4 acetylation. In addition to p53, Maspin is also known as a downstream target gene of PTEN which enhanced Maspin expression through negative regulation of Akt activity by blocking Akt phosphorylation at both T308 and S473 sites (10).

**Figure 3:**
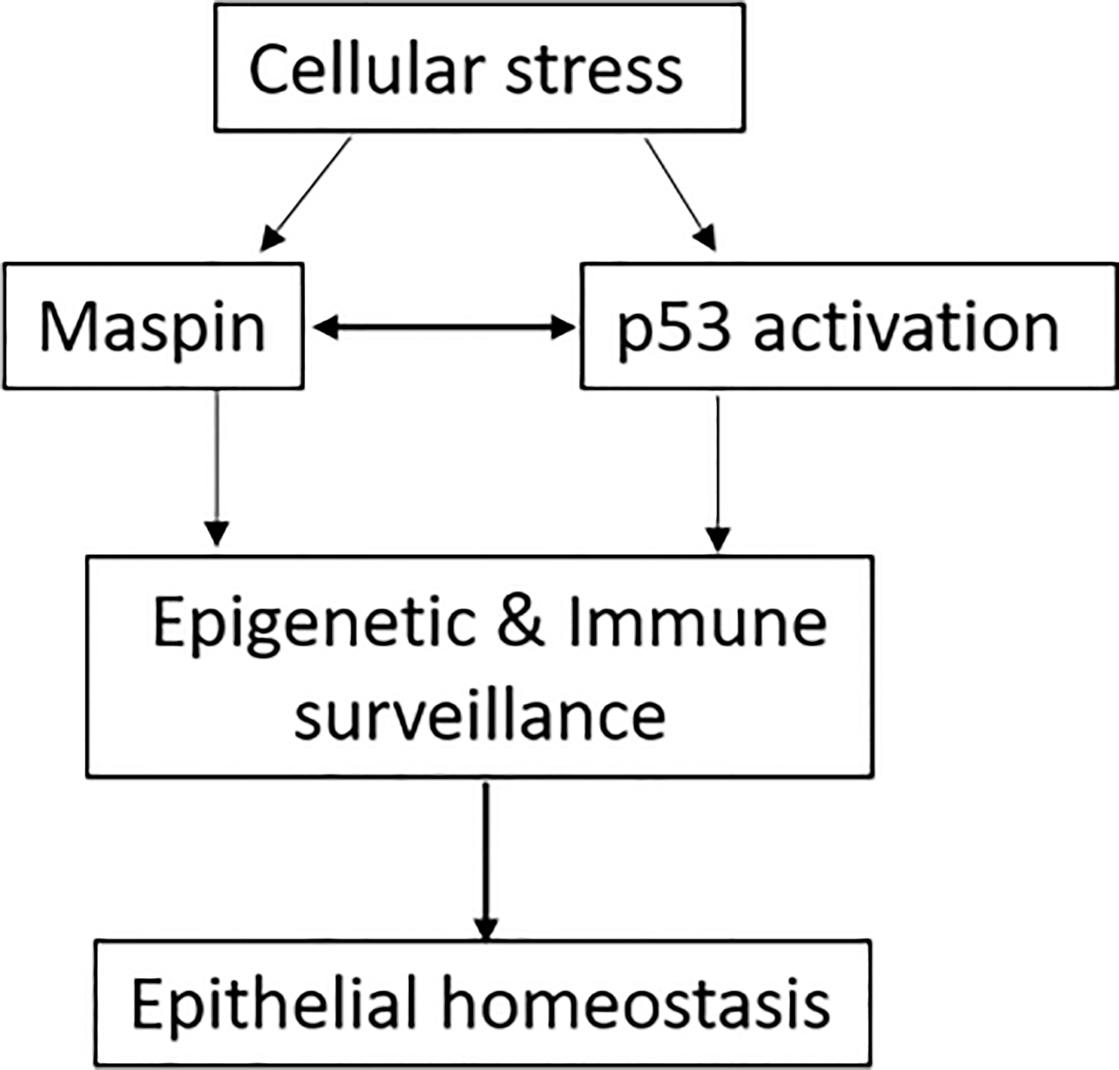
The schematic of Maspin and p53 working hypothesis. Cellular stress promoted the integration of Maspin and p53, in turn arousing host epigenetic and immune surveillance to protect the epithelial digressional process until discharging the cellular stress, consequentially retuning the epithelial homeostasis.

It was evidenced that an abnormal epigenetic regulation was involved in the occurrence and development of cancer (73). Interestingly, HDAC1-mediated deacetylation of p53 attenuated its transcriptional activity and function (74). Thus, as an endogenous inhibitor of HDAC1, Maspin could promote the acetylation of p53 and increase its tumor-suppressive activity theoretically, which was experimentally determined in our laboratory (data available upon request). In addition, both Maspin and p53 were reported to bind to the AR gene promoter, repress AR transcription and expression in prostate cancer cells (9, 24). Taken together, we could propose that, under cellular stress stimulation (e.g., chronic infection/inflammation, oxidative stress, transformation), Maspin expression/activity is augmented which increases acetylation and transcriptional activity of tumor-suppressive p53 by targeting HDAC1. Interactively and sequentially, p53 feedbacks to bind to the Maspin promoter and further increases Maspin transcription and expression. Thus, the tumor-suppressive activities (e.g., immune surveillance, anti-tumorous biology) of both Maspin and p53 were integrated by an HDAC1-mediated interaction and formed a “self-propelling” antitumor mechanism to prevent the digressional process until discharging the cellular stress and consequentially fine-tuning the epithelial homeostasis (**Figure 3**). Thus, by integrating the tumor-suppressive activity of both p53 and Maspin, this cellular stress-aroused Maspin working model was well applicable to explain Maspin’s surveillant role for epithelial homeostasis with its antitumor activity. For instance, in prostate cancer, both Maspin and p53 inhibited the overactivation of AR, which could certainly benefit patient recovery and contribute to the AR-targeted therapy (9, 24). It is noteworthy that epigenetically targeting HDAC1 is a central step in this model, and therefore, HDAC1 is also an applicable target for prostate cancer chemotherapy.

Certainly, it is understandable that the above-proposed “self-propelling” antitumor mechanism functions in a normal physiological epithelial environment to fine-tune and maintain the homeostasis. We also summarized the studies of Maspin-associated molecule/pathways in relation to the status of p53 expression (**Table 1**) and tried to point out that, in a pathological situation of cancer, not only Maspin/p53 interaction but also other molecules were involved/required to compensate for/participate in maintaining the body’s homeostasis.

**Table 1:** The antitumor activity of Maspin-associated molecules/pathways in common human cancers.

Epithelial cancers	Maspin	p53	Pathway	Prognosis/cell fate	References
Prostate cancer	+	NA^*^	Caspase-3	Sensitize to chemotherapy/apoptosis	(53)
	+	NA	p38MAPK	Sensitize to chemotherapy/apoptosis	(75)
	+	NA	Bad, Bax	Sensitize to chemotherapy/apoptosis	(76, 77)
	+	NA	AR	Sensitize to chemotherapy/apoptosis	(9)
	+	NA	AKT, focal adhesion kinase	Sensitize to chemotherapy/apoptosis	(78)
	+	NA	AR-Maspin	Sensitize to chemotherapy/apoptosis	(79)
	+	NA	PTEN/Maspin	Sensitize to chemotherapy/apoptosis	(80)
	+	+	p53-Maspin	Negatively regulate invasion/metastasis	(70, 81)
	+	+	p53, HDAC1, HDAC8	Delay proliferation and migration	(7)
Breast cancer	+	NA	PARP, caspase-3, caspase-8	Apoptosis	(82, 83)
	+	+	NA	Aggressive phenotype Shorter survival	(84)
	+	+	Zinc oxide nanoparticle (ZNP)-Maspin/p53/Bax	Apoptosis, low viability	(8)
	+	+	Smads/p53-Maspin	Inhibition of cell migration	(85)
	+	+	p53-Maspin	Negatively regulate invasion/metastasis	(70, 86, 87)
	+	+	Maspin, p53, bcl2	Apoptosis	(88)
Gastric cancer	+	NA	Caspase3	Apoptosis	(89)
	+	NA	IKKα-Maspin	Mitigate the inflammation, apoptosis	(90)
	+	NA	NA	Lymph node metastasis	(91)
	+	+	p53-nuclear Maspin	Longer survival	(92)
	+	+	p53-Maspin DNA methylation	Decrease the metastasis	(72, 93)
Non-small cell lung cancer cells	+	NA	ERK1/2	Apoptosis	(94)
	+	NA	ATFs-Maspin	Apoptosis, inhibit metastatic dissemination	(95)
	+	+	Nuclear Maspin -p53	Not associated with tumor-specific survival	(96)
	+	+	Maspin, p53	Favorable prognosis	(97)

*NA, not available.

## Conclusion and outlook

Numerous data provided the antitumor activity of Maspin with its surveillant role in retuning epithelial homeostasis, including inhibiting EMT and dedifferentiation, inducing transformed cell re-differentiation, sensitizing the malignant cell to apoptosis induction, and preventing tumorigenesis and restraining tumor progression. This biological anti-tumorous activity involved a very wide range of complex molecular associations and in turn strengthens the host immune surveillance and epithelial homeostasis. Thus, in addition to being a prognostic biomarker of cancer therapy, Maspin has been becoming an attractive subject in the development of an end product of natural antitumor molecule.

As a biomarker of epithelial cells with endogenous HDAC1 inhibitory activity, Maspin could be an ideal substitutional natural molecule for developing an antitumor drug while lowering the side effect induced by synthetic compounds of HDAC inhibitors. In addition, in the light of epigenetic silenced expression of Maspin in tumor, reactivation of Maspin expression by utilizing epigenetic strategy/drugs could produce synergic treatment efficacy and reduce the dosage of chemotherapy. Moreover, it has been reported that the subcellular organelle exosome can be a carrier to transport Maspin protein (98). Thus, it is reasonable to believe that Maspin-engineered exosomes may have a positive impact on prostate cancer treatment.

The extracellular matrix (ECM) is a fundamental and core component of all tissues. ECM can serve as a scaffold for the growth of various epithelial cells and thus affect the function of epithelial cells (99). It was believed that ECM remodeling plays an important role in the inflammatory and immunological milieu for tumorigenesis and progress. Maspin RSL could enhance the adhesion between tumor cells and ECM and subsequently inhibit cell invasion (100). Moreover, Maspin was reported to suppress the invasion of metastatic breast cancer cells by blocking the uPA/uPAR complex (101). Thus, utilizing the bioengineered core structure of Maspin to stabilize the overall environment of ECM could possibly strengthen its role in maintaining epithelial homeostasis and preventing its digression during its development (102).

In summary, we proposed a “self-propelling” antitumor mechanism of Maspin with p53 by inhibition of HDAC1-mediated integrating to retune the epithelial homeostasis. This could direct the research and development for the clinical application of Maspin and p53. In fact, it is a common pathological phenomenon for loss expression of Maspin and/or mutation of p53 in human tumors. Theoretically, the efficiency of individual molecules in clinical application should be associated with another molecule status of expression and activity. Thus, it could be conceivable that transforming Maspin and p53 into clinical applications is a challenging but meaningful work. Moreover, it is required for the clinician to design a strategy carefully prior to its implement and execution.

## Author contributions

This work was conceptualized and designed by ST, JJ, and XL; Writing—original draft preparation was done by ST; review and editing by ZL, XG, and YL; visualization by CW and HC; and finalizing by XL. All authors contributed to the article and approved the submitted version.

## Funding

This review was supported by the National Natural Science Foundation of China (81572741), the Wu Jieping Medical Foundation (320.6750.14120), and the Zhangjiagang Science and Technology support program (ZKS2047). The funding sponsors had no role in the study design, collection, analysis, or interpretation of data or in writing the report.

## Conflict of interest

The authors declare that the review was conducted in the absence of any commercial or financial relationships that could be construed as a potential conflict of interest.

## Publisher’s note

All claims expressed in this article are solely those of the authors and do not necessarily represent those of their affiliated organizations, or those of the publisher, the editors and the reviewers. Any product that may be evaluated in this article, or claim that may be made by its manufacturer, is not guaranteed or endorsed by the publisher.

## References

[1] Zou Z Anisowicz A Hendrix MJ Thor A Neveu M Sheng S . Maspin, a serpin with tumor-suppressing activity in human mammary epithelial cells. Science (1994) 263(5146):526–9. doi: 10.1126/science.8290962 8290962

[2] Gao F Shi HY Daughty C Cella N Zhang M . Maspin plays an essential role in early embryonic development. Development (2004) 131(7):1479–89. doi: 10.1242/dev.01048 14985257

[3] Bodenstine TM Seftor RE Khalkhali-Ellis Z Seftor EA Pemberton PA Hendrix MJ . Maspin: molecular mechanisms and therapeutic implications. Cancer Metastasis Rev (2012) 31(3-4):529–51. doi: 10.1007/s10555-012-9361-0 22752408

[4] Khanaree C Chairatvit K Roytrakul S Wongnoppavich A . Reactive center loop moiety is essential for the maspin activity on cellular invasion and ubiquitin-proteasome level. Oncol Res (2013) 20(9):427–35. doi: 10.3727/096504013X13657689383175 23924927

[5] Al-Ayyoubi M Gettins PG Volz K . Crystal structure of human maspin, a serpin with antitumor properties: reactive center loop of maspin is exposed but constrained. J Biol Chem (2004) 279(53):55540–4. doi: 10.1074/jbc.M409957200 15501821

[6] Ravenhill L Wagstaff L Edwards DR Ellis V Bass R . G-Helix of maspin mediates effects on cell migration and adhesion. J Biol Chem (2010) 285(47):36285–92. doi: 10.1074/jbc.M110.177253 PMC2978556 20837467

[7] Shankar E Pandey M Verma S Abbas A Candamo M Kanwal R . Role of class I histone deacetylases in the regulation of maspin expression in prostate cancer. Mol Carcinog (2020) 59(8):955–66. doi: 10.1002/mc.23214 PMC7436196 32391971

[8] Khorsandi L Farasat M . Zinc oxide nanoparticles enhance expression of maspin in human breast cancer cells. Environ Sci pollut Res Int (2020) 27(30):38300–10. doi: 10.1007/s11356-020-09986-5 32621200

[9] Tang S Lian X Jiang J Cheng H Guo J Huang C . Tumor suppressive maspin-sensitized prostate cancer to drug treatment through negative regulating androgen receptor expression. Front Cell Dev Biol (2020) 8:573820. doi: 10.3389/fcell.2020.573820 33195208 PMC7649228

[10] Lin YH Tsui KH Chang KS Hou CP Feng TH Juang HH . Maspin is a PTEN-upregulated and p53-upregulated tumor suppressor gene and acts as an HDAC1 inhibitor in human bladder cancer. Cancers (Basel) (2019) 12(1):10. doi: 10.3390/cancers12010010 31861435 PMC7016534

[11] Kaplun A Dzinic S Bernardo M Sheng S . Tumor suppressor maspin as a rheostat in HDAC regulation to achieve the fine-tuning of epithelial homeostasis. Crit Rev Eukaryot Gene Expr (2012) 22(3):249–58. doi: 10.1615/CritRevEukarGeneExpr.v22.i3.80 PMC3612174 23140166

[12] Gurzu S Jung I . Subcellular expression of maspin in colorectal cancer: Friend or foe. Cancers (Basel) (2021) 13(3):366. doi: 10.3390/cancers13030366 33498377 PMC7864036

[13] Longhi MT Silva LE Pereira M Magalhaes M Reina J Vitorino FNL . PI3K-AKT, JAK2-STAT3 pathways and cell-cell contact regulate maspin subcellular localization. Cell Commun Signal (2021) 19(1):86. doi: 10.1186/s12964-021-00758-3 34391444 PMC8364028

[14] Huisman C van der Wijst MG Schokker M Blancafort P Terpstra MM Kok K . Re-expression of selected epigenetically silenced candidate tumor suppressor genes in cervical cancer by TET2-directed demethylation. Mol Ther (2016) 24(3):536–47. doi: 10.1038/mt.2015.226 PMC4786921 26686387

[15] Baghel K Kazmi HR Chandra A Raj S Srivastava RN . Significance of methylation status of MASPIN gene and its protein expression in prognosis of gallbladder cancer. Asia Pac J Clin Oncol (2019) 15(5):e120–e5. doi: 10.1111/ajco.13129 30740890

[16] Ramaiah MJ Tangutur AD Manyam RR . Epigenetic modulation and understanding of HDAC inhibitors in cancer therapy. Life Sci (2021) 277:119504. doi: 10.1016/j.lfs.2021.119504 33872660

[17] Weichert W . HDAC expression and clinical prognosis in human malignancies. Cancer Lett (2009) 280(2):168–76. doi: 10.1016/j.canlet.2008.10.047 19103471

[18] Li Y Seto E . HDACs and HDAC inhibitors in cancer development and therapy. Cold Spring Harb Perspect Med (2016) 6(10):a026831. doi: 10.1101/cshperspect.a026831 27599530 PMC5046688

[19] Bernardo MM Meng Y Lockett J Dyson G Dombkowski A Kaplun A . Maspin reprograms the gene expression profile of prostate carcinoma cells for differentiation. Genes Cancer (2011) 2(11):1009–22. doi: 10.1177/1947601912440170 PMC3379563 22737267

[20] Dzinic SH Bernardo MM Oliveira DS Wahba M Sakr W Sheng S . Tumor suppressor maspin as a modulator of host immune response to cancer. Bosn J Basic Med Sci (2015) 15(4):1–6. doi: 10.17305/bjbms.2015.783 PMC4690435 26614844

[21] Chen J Wang L Tang Y Gong G Liu L Chen M . Maspin enhances cisplatin chemosensitivity in bladder cancer T24 and 5637 cells and correlates with prognosis of muscle-invasive bladder cancer patients receiving cisplatin based neoadjuvant chemotherapy. J Exp Clin Cancer Res (2016) 35:2. doi: 10.1186/s13046-015-0282-y 26733306 PMC4702361

[22] Xia W Lau YK Hu MC Li L Johnston DA Sheng S . High tumoral maspin expression is associated with improved survival of patients with oral squamous cell carcinoma. Oncogene (2000) 19(20):2398–403. doi: 10.1038/sj.onc.1203535 10828881

[23] Machtens S Serth J Bokemeyer C Bathke W Minssen A Kollmannsberger C . Expression of the p53 and maspin protein in primary prostate cancer: correlation with clinical features. Int J Cancer (2001) 95(5):337–42. doi: 10.1002/1097-0215(20010920)95:5<337::AID-IJC1059>3.0.CO;2-1 11494236

[24] Alimirah F Panchanathan R Chen J Zhang X Ho SM Choubey D . Expression of androgen receptor is negatively regulated by p53. Neoplasia (2007) 9(12):1152–9. doi: 10.1593/neo.07769 PMC2134911 18084622

[25] Li X Kaplun A Lonardo F Heath E Sarkar FH Irish J . HDAC1 inhibition by maspin abrogates epigenetic silencing of glutathione s-transferase pi in prostate carcinoma cells. Mol Cancer Res (2011) 9(6):733–45. doi: 10.1158/1541-7786.MCR-10-0505 PMC3612175 21622623

[26] Sun Q Zhang K Li H Chen W Liu L Huang G . The overexpression of maspin increases the sensitivity of lung adenocarcinoma drug-resistant cells to docetaxel *in vitro* and in vivo. Ann Transl Med (2020) 8(22):1522. doi: 10.21037/atm-20-7053 33313267 PMC7729325

[27] Katakura H Takenaka K Nakagawa M Sonobe M Adachi M Ito S . Maspin gene expression is a significant prognostic factor in resected non-small cell lung cancer (NSCLC). maspin in NSCLC. Lung Cancer (2006) 51(3):323–8. doi: 10.1016/j.lungcan.2005.10.017 16406136

[28] Matsushige T Sakabe T Umekita Y . Investigation of the subcellular localization-dependent anti- or pro-tumor functions of maspin in human lung adenocarcinoma cell line. Yonago Acta Med (2022) 65(1):44–52. doi: 10.33160/yam.2022.02.006 35221759 PMC8857674

[29] Sopel M Surowiak P Berdowska I . Nuclear maspin expression as a good prognostic factor in human epithelial ovarian carcinoma. Folia Morphol (Warsz) (2010) 69(4):204–12. 21120806

[30] Sakabe T Wakahara M Shiota G Umekita Y . Role of cytoplasmic localization of maspin in promoting cell invasion in breast cancer with aggressive phenotype. Sci Rep (2021) 11(1):11321. doi: 10.1038/s41598-021-90887-z 34059749 PMC8166868

[31] Uchinaka EI Sakabe T Hanaki T Tokuyasu N Sakamoto T Honjo S . Cytoplasmic-only expression of maspin predicts unfavorable prognosis in patients with pancreatic ductal adenocarcinoma. Anticancer Res (2021) 41(5):2543–52. doi: 10.21873/anticanres.15032 33952482

[32] Brabletz S Schuhwerk H Brabletz T Stemmler MP . Dynamic EMT: a multi-tool for tumor progression. EMBO J (2021) 40(18):e108647. doi: 10.15252/embj.2021108647 34459003 PMC8441439

[33] Pastushenko I Blanpain C . EMT transition states during tumor progression and metastasis. Trends Cell Biol (2019) 29(3):212–26. doi: 10.1016/j.tcb.2018.12.001 30594349

[34] Wang N Chang LL . Maspin suppresses cell invasion and migration in gastric cancer through inhibiting EMT and angiogenesis *via* ITGB1/FAK pathway. Hum Cell (2020) 33(3):663–75. doi: 10.1007/s13577-020-00345-7 32409959

[35] Lai Z Yang Y Wang C Yang W Yan Y Wang Z . Circular RNA 0047905 acts as a sponge for microRNA4516 and microRNA1227-5p, initiating gastric cancer progression. Cell Cycle (2019) 18(14):1560–72. doi: 10.1080/15384101.2019.1618122 PMC6619930 31157588

[36] Bircan A Bircan S Kapucuoglu N Songur N Ozturk O Akkaya A . Maspin, VEGF and p53 expression in small biopsies of primary advanced lung cancer and relationship with clinicopathologic parameters. Pathol Oncol Res (2010) 16(4):553–61. doi: 10.1007/s12253-010-9259-5 20349288

[37] Villares GJ Zigler M Dobroff AS Wang H Song R Melnikova VO . Protease activated receptor-1 inhibits the maspin tumor-suppressor gene to determine the melanoma metastatic phenotype. Proc Natl Acad Sci U S A. (2011) 108(2):626–31. doi: 10.1073/pnas.1006886108 PMC3021062 21187389

[38] Zhang M Maass N Magit D Sager R . Transactivation through ets and Ap1 transcription sites determines the expression of the tumor-suppressing gene maspin. Cell Growth Differ (1997) 8(2):179–86. 9040939

[39] Neal CL Henderson V Smith BN McKeithen D Graham T Vo BT . Snail transcription factor negatively regulates maspin tumor suppressor in human prostate cancer cells. BMC Cancer (2012) 12:336. doi: 10.1186/1471-2407-12-336 22857708 PMC3437215

[40] Sharma G Mirza S Parshad R Srivastava A Gupta SD Pandya P . Clinical significance of maspin promoter methylation and loss of its protein expression in invasive ductal breast carcinoma: correlation with VEGF-a and MTA1 expression. Tumour Biol (2011) 32(1):23–32. doi: 10.1007/s13277-010-0087-8 20697987

[41] Li X Yin S Meng Y Sakr W Sheng S . Endogenous inhibition of histone deacetylase 1 by tumor-suppressive maspin. Cancer Res (2006) 66(18):9323–9. doi: 10.1158/0008-5472.CAN-06-1578 16982778

[42] Maass N Biallek M Rosel F Schem C Ohike N Zhang M . Hypermethylation and histone deacetylation lead to silencing of the maspin gene in human breast cancer. Biochem Biophys Res Commun (2002) 297(1):125–8. doi: 10.1016/S0006-291X(02)02136-8 12220518

[43] Cheng H Tang S Lian X Meng H Gu X Jiang J . The differential antitumor activity of 5-Aza-2'-deoxycytidine in prostate cancer DU145, 22RV1, and LNCaP cells. J Cancer (2021) 12(18):5593–604. doi: 10.7150/jca.56709 PMC8364635 34405020

[44] Shilpi A Parbin S Sengupta D Kar S Deb M Rath SK . Mechanisms of DNA methyltransferase-inhibitor interactions: Procyanidin B2 shows new promise for therapeutic intervention of cancer. Chem Biol Interact (2015) 233:122–38. doi: 10.1016/j.cbi.2015.03.022 25839702

[45] Beltran AS Sun X Lizardi PM Blancafort P . Reprogramming epigenetic silencing: artificial transcription factors synergize with chromatin remodeling drugs to reactivate the tumor suppressor mammary serine protease inhibitor. Mol Cancer Ther (2008) 7(5):1080–90. doi: 10.1158/1535-7163.MCT-07-0526 PMC4417684 18483297

[46] He ML Jiang AL Zhang PJ Hu XY Liu ZF Yuan HQ . Identification of androgen-responsive element ARE and Sp1 element in the maspin promoter. Chin J Physiol (2005) 48(3):160–6. 16304843

[47] Zhang M Magit D Sager R . Expression of maspin in prostate cells is regulated by a positive ets element and a negative hormonal responsive element site recognized by androgen receptor. Proc Natl Acad Sci U S A. (1997) 94(11):5673–8. doi: 10.1073/pnas.94.11.5673 PMC20837 9159131

[48] Lin PC Hsieh HY Chu PC Chen CS . Therapeutic opportunities of targeting histone deacetylase isoforms to eradicate cancer stem cells. Int J Mol Sci (2018) 19(7):1939. doi: 10.3390/ijms19071939 30004423 PMC6073995

[49] Tang S Lian X Cheng H Guo J Ni D Huang C . Bacterial lipopolysaccharide augmented malignant transformation and promoted the stemness in prostate cancer epithelial cells. J Inflammation Res (2021) 14:5849–62. doi: 10.2147/JIR.S332943 PMC8590462 34785925

[50] Basu A Paul MK Alioscha-Perez M Grosberg A Sahli H Dubinett SM . Statistical parametrization of cell cytoskeleton reveals lung cancer cytoskeletal phenotype with partial EMT signature. Commun Biol (2022) 5(1):407. doi: 10.1038/s42003-022-03358-0 35501466 PMC9061773

[51] Al-Mamun M Ravenhill L Srisukkham W Hossain A Fall C Ellis V . Effects of noninhibitory serpin maspin on the actin cytoskeleton: A quantitative image modeling approach. Microsc Microanal (2016) 22(2):394–409. doi: 10.1017/S1431927616000520 26906065

[52] Dzinic SH Bernardo MM Li X Fernandez-Valdivia R Ho YS Mi QS . An essential role of maspin in embryogenesis and tumor suppression. Cancer Res (2017) 77(4):886–96. doi: 10.1158/0008-5472.CAN-16-2219 PMC5313336 27923833

[53] Tahmatzopoulos A Sheng S Kyprianou N . Maspin sensitizes prostate cancer cells to doxazosin-induced apoptosis. Oncogene (2005) 24(34):5375–83. doi: 10.1038/sj.onc.1208684 PMC2274915 16007219

[54] Zhang W Shi HY Zhang M . Maspin overexpression modulates tumor cell apoptosis through the regulation of bcl-2 family proteins. BMC Cancer (2005) 5:50. doi: 10.1186/1471-2407-5-50 15907209 PMC1156873

[55] Schaefer JS Zhang M . Targeting maspin in endothelial cells to induce cell apoptosis. Expert Opin Ther Targets (2006) 10(3):401–8. doi: 10.1517/14728222.10.3.401 16706680

[56] Bernardo MM Kaplun A Dzinic SH Li X Irish J Mujagic A . Maspin expression in prostate tumor cells averts stemness and stratifies drug sensitivity. Cancer Res (2015) 75(18):3970–9. doi: 10.1158/0008-5472.CAN-15-0234 PMC4573892 26208903

[57] Mao X Xu J Wang W Liang C Hua J Liu J . Crosstalk between cancer-associated fibroblasts and immune cells in the tumor microenvironment: new findings and future perspectives. Mol Cancer (2021) 20(1):131. doi: 10.1186/s12943-021-01428-1 34635121 PMC8504100

[58] Cassetta L Fragkogianni S Sims AH Swierczak A Forrester LM Zhang H . Human tumor-associated macrophage and monocyte transcriptional landscapes reveal cancer-specific reprogramming, biomarkers, and therapeutic targets. Cancer Cell (2019) 35(4):588–602 e10. doi: 10.1016/j.ccell.2019.02.009 30930117 PMC6472943

[59] Jian Y Yang K Sun X Zhao J Huang K Aldanakh A . Current advance of immune evasion mechanisms and emerging immunotherapies in renal cell carcinoma. Front Immunol (2021) 12:639636. doi: 10.3389/fimmu.2021.639636 33767709 PMC7985340

[60] Philip M Schietinger A . CD8(+) T cell differentiation and dysfunction in cancer. Nat Rev Immunol (2022) 22(4):209–23. doi: 10.1038/s41577-021-00574-3 PMC9792152 34253904

[61] Joglekar AV Leonard MT Jeppson JD Swift M Li G Wong S . T Cell antigen discovery *via* signaling and antigen-presenting bifunctional receptors. Nat Methods (2019) 16(2):191–8. doi: 10.1038/s41592-018-0304-8 PMC6755906 30700902

[62] Besgen P Trommler P Vollmer S Prinz JC . Ezrin, maspin, peroxiredoxin 2, and heat shock protein 27: potential targets of a streptococcal-induced autoimmune response in psoriasis. J Immunol (2010) 184(9):5392–402. doi: 10.4049/jimmunol.0903520 20363977

[63] Wang Y Sun L Song Z Wang D Bao Y Li Y . Maspin inhibits macrophage phagocytosis and enhances inflammatory cytokine production *via* activation of NF-kappaB signaling. Mol Immunol (2017) 82:94–103. doi: 10.1016/j.molimm.2016.12.021 28064070

[64] Li L Wang X . Identification of gastric cancer subtypes based on pathway clustering. NPJ Precis Oncol (2021) 5(1):46. doi: 10.1038/s41698-021-00186-z 34079012 PMC8172826

[65] Girish GV Bhattacharya G Sinha AK . The role of insulin dependent NO synthesis in the impaired production of maspin in human breast cancer. J Cancer Res Clin Oncol (2006) 132(6):389–98. doi: 10.1007/s00432-006-0087-7 PMC12161087 16491398

[66] Ganguly Bhattacharjee K Bhattacharyya M Halder UC Jana P Sinha AK . The role of neutrophil estrogen receptor status on maspin synthesis *via* nitric oxide production in human breast cancer. J Breast Cancer (2012) 15(2):181–8. doi: 10.4048/jbc.2012.15.2.181 PMC3395741 22807935

[67] Tanaka A Sakaguchi S . Regulatory T cells in cancer immunotherapy. Cell Res (2017) 27(1):109–18. doi: 10.1038/cr.2016.151 PMC5223231 27995907

[68] Tan W Zhang W Strasner A Grivennikov S Cheng JQ Hoffman RM . Tumour-infiltrating regulatory T cells stimulate mammary cancer metastasis through RANKL-RANK signalling. Nature (2011) 470(7335):548–53. doi: 10.1038/nature09707 PMC3166217 21326202

[69] Barkat MA Harshita Ahmad J Khan MA Beg S Ahmad FJ . Insights into the targeting potential of thymoquinone for therapeutic intervention against triple-negative breast cancer. Curr Drug Targets (2018) 19(1):70–80. doi: 10.2174/1389450118666170612095959 28606050

[70] Zou Z Gao C Nagaich AK Connell T Saito S Moul JW . p53 regulates the expression of the tumor suppressor gene maspin. J Biol Chem (2000) 275(9):6051–4. doi: 10.1074/jbc.275.9.6051 10692390

[71] Hafner A Bulyk ML Jambhekar A Lahav G . The multiple mechanisms that regulate p53 activity and cell fate. Nat Rev Mol Cell Biol (2019) 20(4):199–210. doi: 10.1038/s41580-019-0110-x 30824861

[72] Gurzu S Jung I Sugimura H Stefan-van Staden RI Yamada H Natsume H . Maspin subcellular expression in wild-type and mutant TP53 gastric cancers. World J Gastrointest Oncol (2020) 12(7):741–55. doi: 10.4251/wjgo.v12.i7.741 PMC7428795 32864042

[73] Dawson MA Kouzarides T . Cancer epigenetics: from mechanism to therapy. Cell (2012) 150(1):12–27. doi: 10.1016/j.cell.2012.06.013 22770212

[74] Gu W Luo J Brooks CL Nikolaev AY Li M . Dynamics of the p53 acetylation pathway. Novartis Found Symp (2004) 259:197–205. discussion -7, 23-5. doi: 10.1002/0470862637.ch14 15171255

[75] Li X Chen D Yin S Meng Y Yang H Landis-Piwowar KR . Maspin augments proteasome inhibitor-induced apoptosis in prostate cancer cells. J Cell Physiol (2007) 212(2):298–306. doi: 10.1002/jcp.21102 17458898

[76] Cheng WL Huang CY Tai CJ Chang YJ Hung CS . Maspin enhances the anticancer activity of curcumin in hormone-refractory prostate cancer cells. Anticancer Res (2018) 38(2):863–70. doi: 10.21873/anticanres.12295 29374713

[77] Liu J Yin S Reddy N Spencer C Sheng S . Bax mediates the apoptosis-sensitizing effect of maspin. Cancer Res (2004) 64(5):1703–11. doi: 10.1158/0008-5472.CAN-03-2568 14996730

[78] McKenzie S Sakamoto S Kyprianou N . Maspin modulates prostate cancer cell apoptotic and angiogenic response to hypoxia *via* targeting AKT. Oncogene (2008) 27(57):7171–9. doi: 10.1038/onc.2008.321 PMC2725761 18931702

[79] Liu W Zhou J Geng G Shi Q Sauriol F Wu JH . Antiandrogenic, maspin induction, and antiprostate cancer activities of tanshinone IIA and its novel derivatives with modification in ring a. J Med Chem (2012) 55(2):971–5. doi: 10.1021/jm2015292 22175694

[80] Gan Y Chen Q Lei Y . Regulation of paclitaxel sensitivity in prostate cancer cells by PTEN/maspin signaling. Oncol Lett (2017) 14(4):4977–82. doi: 10.3892/ol.2017.6793 PMC5649693 29085510

[81] Machtens S Kuczyk M Serth J Jonas U . P53 regulated maspin protein expression determines recurrence-free survival of patients with localised prostate cancer. Prostate Cancer Prostatic Dis (2000) 3(S1):S27. doi: 10.1038/sj.pcan.4500453 12497136

[82] Jiang N Meng Y Zhang S Mensah-Osman E Sheng S . Maspin sensitizes breast carcinoma cells to induced apoptosis. Oncogene (2002) 21(26):4089–98. doi: 10.1038/sj.onc.1205507 12037665

[83] Latha K Zhang W Cella N Shi HY Zhang M . Maspin mediates increased tumor cell apoptosis upon induction of the mitochondrial permeability transition. Mol Cell Biol (2005) 25(5):1737–48. doi: 10.1128/MCB.25.5.1737-1748.2005 PMC549349 15713631

[84] Lee MJ Suh CH Li ZH . Clinicopathological significance of maspin expression in breast cancer. J Korean Med Sci (2006) 21(2):309–14. doi: 10.3346/jkms.2006.21.2.309 PMC2734010 16614520

[85] Wang SE Narasanna A Whitell CW Wu FY Friedman DB Arteaga CL . Convergence of p53 and transforming growth factor beta (TGFbeta) signaling on activating expression of the tumor suppressor gene maspin in mammary epithelial cells. J Biol Chem (2007) 282(8):5661–9. doi: 10.1074/jbc.M608499200 PMC4015524 17204482

[86] Oshiro MM Watts GS Wozniak RJ Junk DJ Munoz-Rodriguez JL Domann FE . Mutant p53 and aberrant cytosine methylation cooperate to silence gene expression. Oncogene (2003) 22(23):3624–34. doi: 10.1038/sj.onc.1206545 12789271

[87] Maekawa T Sano Y Shinagawa T Rahman Z Sakuma T Nomura S . ATF-2 controls transcription of maspin and GADD45 alpha genes independently from p53 to suppress mammary tumors. Oncogene (2008) 27(8):1045–54. doi: 10.1038/sj.onc.1210727 17700520

[88] Prasad CP Rath G Mathur S Bhatnagar D Ralhan R . Expression analysis of maspin in invasive ductal carcinoma of breast and modulation of its expression by curcumin in breast cancer cell lines. Chem Biol Interact (2010) 183(3):455–61. doi: 10.1016/j.cbi.2009.11.019 19944674

[89] Wang MC Yang YM Li XH Dong F Li Y . Maspin expression and its clinicopathological significance in tumorigenesis and progression of gastric cancer. World J Gastroenterol (2004) 10(5):634–7. doi: 10.3748/wjg.v10.i5.634 PMC4716899 14991928

[90] Wang N Chang LL . The potential function of IKKalpha in gastric precancerous lesion *via* mediating maspin. Tissue Cell (2020) 65:101349. doi: 10.1016/j.tice.2020.101349 32746986

[91] Terashima M Maesawa C Oyama K Ohtani S Akiyama Y Ogasawara S . Gene expression profiles in human gastric cancer: expression of maspin correlates with lymph node metastasis. Br J Cancer (2005) 92(6):1130–6. doi: 10.1038/sj.bjc.6602429 PMC2361928 15770218

[92] Lee DY Park CS Kim HS Kim JY Kim YC Lee S . Maspin and p53 protein expression in gastric adenocarcinoma and its clinical applications. Appl Immunohistochem Mol Morphol (2008) 16(1):13–8. doi: 10.1097/PAI.0b013e31802c4f21 18091326

[93] Ito R Nakayama H Yoshida K Oda N Yasui W . Loss of maspin expression is associated with development and progression of gastric carcinoma with p53 abnormality. Oncol Rep (2004) 12(5):985–90. doi: 10.3892/or.12.5.985 15492782

[94] Lin K Yang R Zheng Z Zhou Y Geng Y Hu Y . Sulforaphane-cysteine-induced apoptosis *via* phosphorylated ERK1/2-mediated maspin pathway in human non-small cell lung cancer cells. Cell Death Discovery (2017) 3:17025. doi: 10.1038/cddiscovery.2017.25 28690874 PMC5494314

[95] Beltran AS Blancafort P . Reactivation of MASPIN in non-small cell lung carcinoma (NSCLC) cells by artificial transcription factors (ATFs). Epigenetics (2011) 6(2):224–35. doi: 10.4161/epi.6.2.13700 PMC3278788 20948306

[96] Woenckhaus M Bubendorf L Dalquen P Foerster J Blaszyk H Mirlacher M . Nuclear and cytoplasmic maspin expression in primary non-small cell lung cancer. J Clin Pathol (2007) 60(5):483–6. doi: 10.1136/jcp.2005.033407 PMC1994526 16698957

[97] Nakagawa M Katakura H Adachi M Takenaka K Yanagihara K Otake Y . Maspin expression and its clinical significance in non-small cell lung cancer. Ann Surg Oncol (2006) 13(11):1517–23. doi: 10.1245/s10434-006-9030-z 17009165

[98] Dean I Dzinic SH Bernardo MM Zou Y Kimler V Li X . The secretion and biological function of tumor suppressor maspin as an exosome cargo protein. Oncotarget (2017) 8(5):8043–56. doi: 10.18632/oncotarget.13302 PMC5352381 28009978

[99] Englund JI Ritchie A Blaas L Cojoc H Pentinmikko N Dohla J . Laminin alpha 5 regulates mammary gland remodeling through luminal cell differentiation and Wnt4-mediated epithelial crosstalk. Development (2021) 148(12):dev199281. doi: 10.1242/dev.199281 34128985 PMC8254867

[100] Ngamkitidechakul C Warejcka DJ Burke JM O'Brien WJ Twining SS . Sufficiency of the reactive site loop of maspin for induction of cell-matrix adhesion and inhibition of cell invasion. conversion of ovalbumin to a maspin-like molecule. J Biol Chem (2003) 278(34):31796–806. doi: 10.1074/jbc.M302408200 12799381

[101] Amir S Margaryan NV Odero-Marah V Khalkhali-Ellis Z Hendrix MJ . Maspin regulates hypoxia-mediated stimulation of uPA/uPAR complex in invasive breast cancer cells. Cancer Biol Ther (2005) 4(4):400–6. doi: 10.4161/cbt.4.4.1617 PMC3175738 15846059

[102] Yin S Li X Meng Y Finley RL Jr. Sakr W Yang H . Tumor-suppressive maspin regulates cell response to oxidative stress by direct interaction with glutathione s-transferase. J Biol Chem (2005) 280(41):34985–96. doi: 10.1074/jbc.M503522200 16049007

